# The 14-3-3η chaperone protein promotes antiviral innate immunity via facilitating MDA5 oligomerization and intracellular redistribution

**DOI:** 10.1371/journal.ppat.1007582

**Published:** 2019-02-11

**Authors:** Jhih-Pu Lin, Yu-Kuan Fan, Helene Minyi Liu

**Affiliations:** 1 Graduate Institute of Biochemistry and Molecular Biology, College of Medicine, National Taiwan University, Taipei City, Taiwan; 2 Department of Laboratory Medicine, National Taiwan University Hospital, Taipei City, Taiwan; Emory University, UNITED STATES

## Abstract

MDA5 belongs to the RIG-I-like receptor family and plays a non-redundant role in recognizing cytoplasmic viral RNA to induce the production of type I IFNs. Upon RNA ligand stimulation, we observed the redistribution of MDA5 from the cytosol to mitochondrial membrane fractions. However, the molecular mechanisms of MDA5 activation remain less understood. Here we show that 14-3-3η is an essential accessory protein for MDA5-dependent type I IFN induction. We found that several 14-3-3 isoforms may interact with MDA5 through the CARDs (N-MDA5), but 14-3-3η was the only isoform that could enhance MDA5-dependent IFNβ promoter activities in a dose-dependent manner. Knock-down of 14-3-3η in Huh7 cells impaired and delayed the kinetics of MDA5 oligomerization, which is a critical step for MDA5 activation. Consequently, the MDA5-dependent IFNβ promoter activities as well as IFNβ mRNA expression level were also decreased in the 14-3-3η knocked-down cells. We also demonstrated that 14-3-3η is essential in boosting the activation of MDA5-dependent antiviral innate immunity during viral infections. In conclusion, our results uncover a novel function of 14-3-3η to promote the MDA5-dependent IFNβ induction pathway by reducing the immunostimulatory potential of viral dsRNA within MDA5 activation signaling pathway.

## Author summary

In this study, we utilized biochemistry and molecular biology approaches to defines the molecular mechanisms by which melanoma differentiation-associated protein 5 (MDA5), a cytoplasmic RNA helicase and pattern recognition receptor molecule, is regulated by 14-3-3η to govern its innate immune signaling activity. During viral infection RIG-I-like receptors (RLRs), including MDA5, play essential roles in initiating type I interferon signaling pathway and preventing virus infection or replication in host cells. Besides, the establishment of well functional adaptive immune response to viruses is depending on the timely activation of innate immune antiviral signaling pathway. Our results suggested that the activation of MDA5 is promoted by the chaperone protein 14-3-3η. The lack of 14-3-3η in host cells leads to the kinetically-delayed oligomerization of MDA5, which is a key steps of the activation of MDA5-mediated anti-viral signaling pathway. These findings reveal a novel component which participating in the control system of MDA5-dependent signaling pathway. Viral proteins which antagonize 14-3-3η to impair MDA5-dependent antiviral signaling may be suitable targets for antiviral therapy or be modified to generate potential vaccine strains.

## Introduction

Among the RIG-I-like Receptor (RLR) family, RIG-I and MDA5 share a number of structural similarities, and both of them include three distinct domains. The N-terminus caspase activation and recruitment domains (CARDs) function as the activation domain to directly interact with the CARD of downstream adaptor protein MAVS, of which interaction is critical for the activation of the type I interferon signaling pathway [1–4]. The molecular mechanisms of RIG-I activation have been extensively studied in the past decade. Once bound on the RNA with 5’-triphosphate (5’-ppp) or short dsRNA, a conformational change of RIG-I will occur, and the C-terminus repressor domain (RD) of RIG-I will release the CARDs for interactions with accessory proteins for redistribution and then interaction with downstream signaling molecules [5, 6]. We have previously identified that activated RIG-I will then redistribute to the mitochondrion-associated membrane (MAM) through interaction with mitochondrial chaperone protein 14-3-3ε via the CARDs of RIG-I [7]. The interaction between RIG-I and MAVS at MAM is critical for triggering IFNβ induction [8]. MDA5 activation, however, is not as well-understood as RIG-I. MDA5-mediated antiviral signaling has been shown important in the clearance of flavivirus, picornavirus, paramyxovirus, and reovirus infections [9]. It has been shown that MDA5 has an essential and non-redundant role in detecting RNA virus infection [10]. When viral long dsRNA appears in the cytoplasm, MDA5 can recognize with these long dsRNA by its helicase and C-terminal domains [11]. The interaction between MDA5 to the long dsRNA will then cooperatively form a tandem MDA5 filament along the dsRNA, and the CARDs of MDA5 will then oligomerize and interact with MAVS to trigger antiviral signaling pathway [12]. Finally, this signaling pathway leads to the phosphorylation and activation of transcription factors, such as interferon regulatory factor 3 (IRF3), interferon regulatory factor 7 (IRF7), and nuclear factor κ-light-chain-enhancer of activated B cells (NF-κB) to induce interferon β (IFNβ) and a set of other antiviral genes to restrict virus replication [13–15].

To establish successful infections, viruses have evolved viral components antagonize the host innate immune system. For instance, dengue virus NS3 protein uses a protease-independent phosphomimetic-based mechanism to retain the essential chaperone protein for RIG-I activation, 14-3-3ε, in the cytosol and lead to the attenuation of IFNβ induction in the infected cells [16]. Successful type I IFN induction requires both effective recognition and activation of the receptors as well as complete downstream signaling transduction. The race between the proper establishment of host antiviral state and the virus antagonism at early infection is critical to determine the infection outcome [17].

The 14-3-3 proteins are conserved in most animals and plants. In mammalian cells, there are 7 isoforms known as 14-3-3β (α), 14-3-3γ, 14-3-3ε, 14-3-3η, 14-3-3σ, 14-3-3θ (τ) and 14-3-3ζ (δ) [18, 19]. There are tremendous amount of intracellular proteins, and the intracellular localization of each proteins serves as a regulatory event during cell cycle control, signaling transduction, cell programmed death, protein trafficking, and etcetera [20–23]. Each isoforms of the 14-3-3 family may form homo- or hetero-dimers to serve as chaperone proteins and relocalize their target proteins from one intracellular compartment to another among cytoplasm, cellular membrane, endoplasmic reticulum (ER), mitochondrion, or the nucleus [7]. In most cases, 14-3-3 chaperone proteins bind onto the target proteins at the original localizations, and upon certain stimulations or post-translational modification of the protein, the 14-3-3 proteins will then bring their target proteins to a specific sites where the proteins can properly function [20, 24, 25].

Previous studies have shown that the expression level of 14-3-3 isoforms were upregulated during virus infection, and overexpression of 14-3-3 protein could attenuate the production of virus particles [26–28]. Also, we previously identified that the redistribution of RIG-I from the cytosol to a membrane fraction upon ligand recognition was controlled by 14-3-3ε [7]. A followed-up study showed that dengue virus (DENV) NS3 protein uses a phosphomimetic-based mechanism to occupy 14-3-3ε to restrict RIG-I activation in the DENV-infected cells [16], suggesting that 14-3-3 family serve as an important regulator in the type I IFN induction pathway. In this study, we anticipated to further understand the molecular mechanisms of MDA5 activation during virus infection or dsRNA stimulation. We have identified that among the 3 isoforms of 14-3-3 proteins that binds to MDA5, 14-3-3η specifically promotes MDA5 activation during viral infections and/or under poly(I:C) stimulation. We found that in the presence of 14-3-3η, MDA5 activation obtained a higher kinetics in both MDA5 oligomerization and MDA5-mediated type I IFN induction, when compared to those in the 14-3-3 knock-down conditions. Our studies reveal that 14-3-3η is the crucial chaperone protein for MDA5-dependent signaling to innate antiviral immunity.

## Materials and methods

### Cells

Human embryonic kidney cell lines, HEK293 and HEK293T (from ATCC), and human hepatoma cell line, Huh7 (obtained from Dr. Michael Gale Jr., University of Washington), were maintained in Dulbecco’s modified Eagle medium (DMEM) supplemented with 10% fetal bovine serum (FBS). Huh7 Non-Targeting Vector (NTV) cells, Huh7 RIG-I knock-down (K/D) cells, Huh7 14-3-3η K/D cells were first transfected with pSUPER.retro.shRNA plasmids (Oligoengine) which contain the shRNA sequences for targeting genes respectively and subsequently selected and maintained in DMEM supplemented with 10% FBS and 1 μg/mL puromycin.

### Viruses infection and poly(I:C) stimulation

Huh7, Huh7 NTV, Huh7 RIG-I K/D, Huh7 14-3-3η K/D cells were infected with Sendai virus (SeV) at a concentration of 200 HA unit/mL in DMEM supplemented with 10% FBS at 37°C. For encephalomyocarditis virus (EMCV) infection, cells were first washed with PBS twice, then were incubated with virus with designated MOI in serum-free medium for 1 hour at 37°C. The cells were rinsed by PBS twice and incubated in DMEM supplemented with 2% FBS post virus solution absorption. For poly(I:C) stimulation, cells were transfected with high molecular weight (HMW) poly(I:C) (Invivogen) by TransIT-mRNA reagent (Mirus) according to manufacturer’s instructions.

### Plasmids and constructs

To generate FLAG-tagged MDA5, MDA5 cDNA sequence was amplified and cloned into pEFTak vector plasmid with an N-terminus FLAG tag. FLAG-N-MDA5 and FLAG-C-MDA5 which contained MDA5 amino acids 1–205, 205–1025 respectively were amplified from full-length MDA5 cDNA sequence and sub-cloned into pEFTak plasmids. FLAG-N-MDA5 S88A and S88D were generated by site-directed mutagenesis with QuikChange Lightning Site-Directed Mutagenesis Kit (Agilent). The detailed experimental procedures were according to manufacturer’s instructions. myc-tagged 14-3-3 isoforms expression constructs were described previously [7]. shRNA constructs targeting 14-3-3η was designed by cloning a hairpin sequence which targeting at human 14-3-3η gene 5’-UTR region into pSUPER.retro.shRNA plasmids.

### Dual luciferase IFNβ reporter assay

Dual luciferase assays to measure Interferon β promoter activity were conducted as described (Saito et al., 2008).

### Immunoblot analysis and immunoprecipitation

Cells were lysed in ice-cold RIPA buffer (50 mM Tris-Cl pH 7.5, 150 mM NaCl, 5 mM EDTA, 1% NP-40, 0.5% sodium deoxycholate, 0.1% SDS) in the presence of Protease Inhibitor Cocktail (Roche) for 10 min. Lysates were clarified by centrifugation and incubated with 2 μg of antibodies for 16 h followed by Protein A/G agarose for 1 h at 4°C. The immunocomplexes were washed 3 times with cold RIPA buffer and resuspended in 15 μl of 2 × SDS sample buffer for SDS-PAGE. Commercial antibodies used in this study were: FLAG-tag mAb (Sigma-Aldrich, F3165), c-myc tag (BETHYL, A190-205A), GAPDH (GeneTex, GTX100118), MDA5 (Enzo, ALX-210-935), RIG-I (AdipoGen, AG-20B-0009), 14-3-3 Family Antibody Sampler Kit (Cell Signaling Technology, #9769), VDAC1 (Abcam, ab15895), ACSL4/FACL4 (OriGENE, TA324720), MAVS/IPS-1 (Enzo, ALX-210-929-C100), Tubulin (Cell Signaling Technology, #2128).

### Semi-denaturing detergent agarose gel electrophoresis (SDD-AGE)

Cells were first lysed with Triton X-100 lysis buffer (0.5% Triton X-100, 50 mM Tris pH7.5, 150 mM NaCl, 10% glycerol) supplemented with protease inhibitor (*Roche*) at 4°C for 20 minutes and followed by centrifugation at 13300 rpm for the clearance of lysates. Equal amounts of total proteins for each sample were incubated with 4X SDD-AGE sample buffer (2X TBE, 4% glycerol, 8% SDS) at room temperature for 10 minutes. Samples were subsequently separated in 1.5% agarose gel which contained 0.1% SDS with 1X running buffer (1X TBE, 0.1% SDS) at 80V, 4°C for 90 minutes. Proteins were transferred to NC membrane and blocked in TBST buffer with 5% non-fat milk.

### Membrane fractionation/ cell fractionation assay

Cells were trypsinized and collected in 1.5 mL microtubes for further steps to fractionate the cells. In brief, cells were first suspended in 1X cytosol fraction buffer (BioVison) and incubated for 10 minutes on ice. Cells suspension was then homogenized by using G21 needles pipetting up and down for 20 times and followed by centrifugation at 700g for 10 minutes to precipitate the remaining unsolved components. Supernatants were subsequently centrifuged at 10000g for 30 minutes to separate cytosol and mitochondria-MAM fractions. The pellet of mitochondria-MAM fraction was re-suspended in 1X mitochondria fraction buffer.

### Statistical analysis

All the experiments were repeated at least three times, and the results were presented as mean ± standard deviation (SD). Data were analyzed by two-tailed Student’s t-test, *p<0.05, **p<0.01, ***P<0.001.

## Results

### The redistribution of signaling-active MDA5 to the mitochondria and/or mitochondria-associated-membrane (MAM) fractions

**Fig 1:**
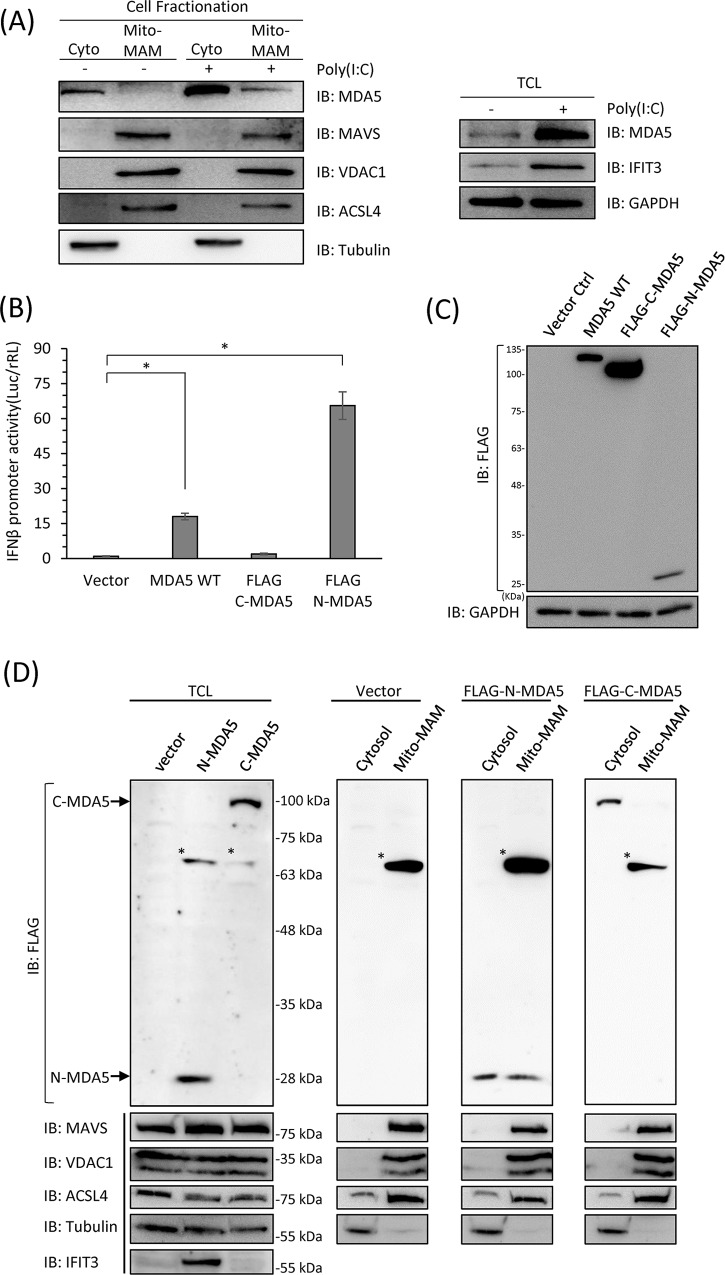
MDA5 redistributes to the mitochondria and/or the mitochondria-associated membrane fraction upon activation. (A) Huh7 cells were mock-treated or stimulated with 1 μg/mL poly(I:C) for 18 hours. The protein levels of IFIT3 was increased in poly(I:C) transfected cell as a positive control. Cell lysates were then fractionated into cytosol and mitochondria-MAM (Mito-MAM) fractions, and the endogenous MDA5 distribution was detected by immunoblotting. Membrane fractions were validated with markers of mitochondria-associated membrane (MAM) (ACSL4) and mitochondria (VDAC1). Tubulin serves as the cytosol marker to show the efficiency of membrane fractionation. (B) Huh7 WT cells were transfected with FLAG-tagged full-length, N-terminal or C-terminal portion of MDA5 for 48 hours. The IFNβ promoter activities were evaluated by dual luciferase assay. *, p<0.05. (C) Protein expression levels of (B) were detected by immunoblotting. (D) Huh7 WT cells were transfected with FLAG-tagged N-MDA5 or C-MDA5 for 48 hours and subjected to fractionation into cytosol and mito-MAM fractions. The distribution of N-MDA5 and C-MDA5 were determined by immunoblotting. *: non-specific bands.

To assess the distribution of MDA5, we conducted mitochondria fractionation of human hepatoma (Huh7) cell extracts to separate mitochondria as well as mitochondria-associated-membrane from the cytosolic cell compartments for analysis of protein localization. Huh7 cells were stimulated with poly(I:C) to induce MDA5 activation. The increased protein levels of both MDA5 and IFIT3 were determined by immunoblotting to confirm the effects of poly(I:C) stimulations (Fig 1A). These cells were then subjected to the fractionation assay. We confirmed the mitochondria-MAM (Mito-MAM) and the cytosolic (Cyto) fractions were well-separated by detecting the distribution of MAVS, the mitochondria marker VDAC1, the MAM marker ACSL4, and the cytosol maker tubulin by immunoblotting (Fig 1A). We found that the endogenous MDA5 was retained in the cytosolic fraction in the uninfected cells, and would be relocalized to the Mito-MAM fraction upon poly(I:C) stimulation (Fig 1A).

In order to determine which domain of MDA5 is responsible for its relocalization, we ectopically expressed wildtype (WT) MDA5, N-MDA5 or C-MDA5 in Huh7 cells and evaluated the distribution of these constructs (S1A Fig). We first confirmed that ectopic expression of full-length MDA5 alone could induce IFNβ promoter activity when compared to the vector control (Fig 1B). The expression of FLAG-tagged N-MDA5, which contains only 2 CARDs of MDA5 (S1A Fig), led to the highest activation of IFNβ promoter activity although the protein level detected by immunoblotting showed that FLAG-N-MDA5 expression was the lowest among the three (Fig 1B and 1C). The expression of FLAG-C-MDA5 did not induce IFNβ promoter activity (Fig 1B and 1C).

We next assessed the distribution of FLAG-N-MDA5 and FLAG-C-MDA5 by Mito-MAM fractionation assay. We ectopically expressed FLAG-N-MDA5 and FLAG-C-MDA5 in Huh7 cells and fractionated cell lysates into cytosol or Mito-MAM fractions. The fractionation competence was analyzed by detecting the distribution of MAVS, VDAC1, ACSL4, and tubulin by immunoblotting (Fig 1D and S1B Fig). FLAG-N-MDA5, the constitutively active MDA5 mutant, could distribute in both cytosol and mitochondria-MAM fractions, whereas FLAG-C-MDA5 was only detected in the cytosol (Fig 1D). The expression of IFIT3 in the total cell lysate suggested the induction of type I IFN by ectopic expression of FLAG-N-MDA5 (Fig 1D). These results indicated that similar to RIG-I, activated MDA5 was redistributed from the cytosol to the Mito-MAM fraction, and this redistribution was governed by the N-terminal CARDs of MDA5.

### 14-3-3η interacts with and facilitates the activation of MDA5

**Fig 2:**
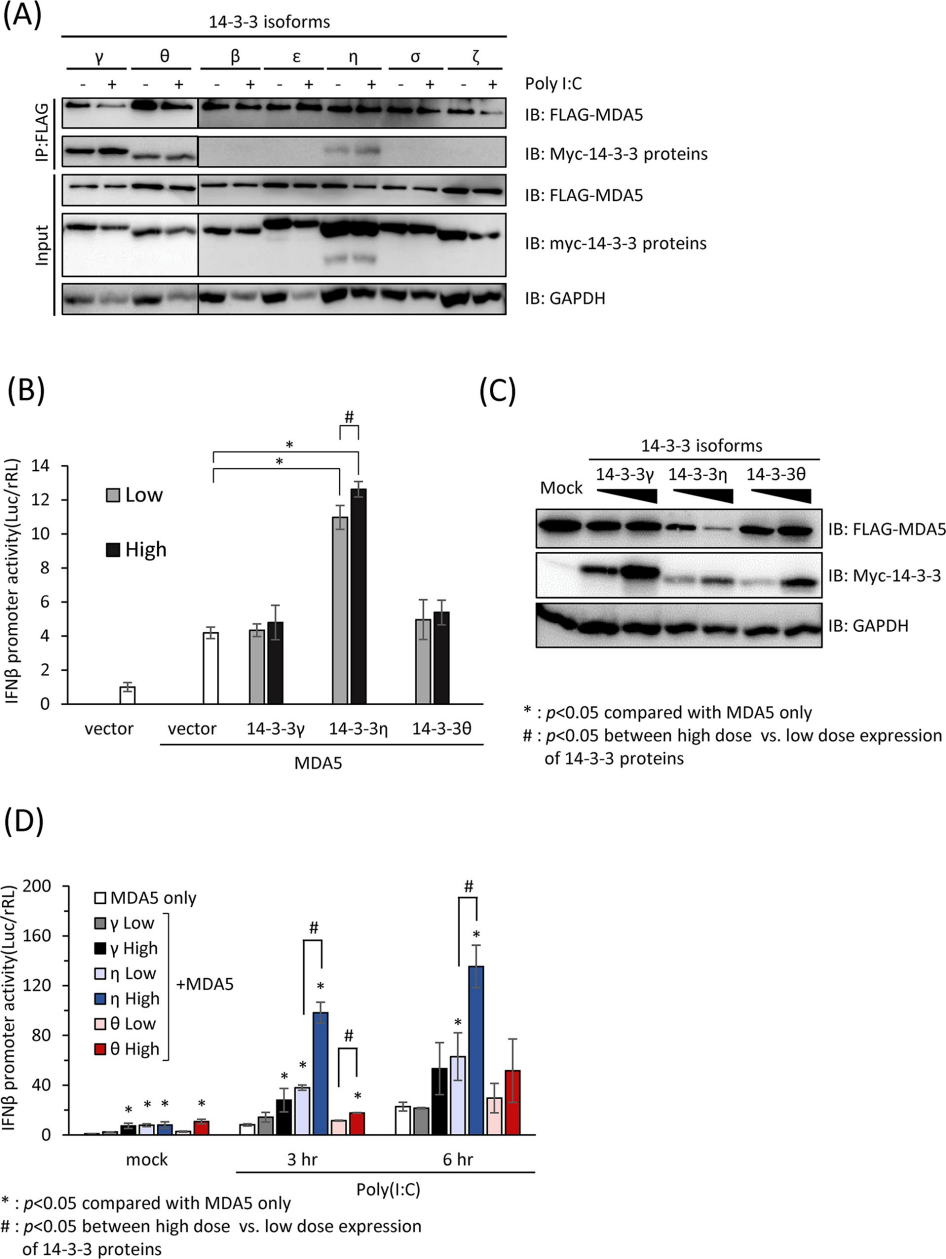
14-3-3η interacts with MDA5 and promotes MDA5-dependent antiviral signaling pathway. (A) Co-immunoprecipitation assay of MDA5 and various 14-3-3 isoforms. Huh7 cells were transfected with FLAG-tagged full-length MDA5 with different isoforms of myc-tagged 14-3-3 proteins and subsequently stimulated with 1μg/mL poly (I:C) for 6 hours or leaved unstimulated (mock). Cell lysates were then subject to anti-FLAG immunoprecipitation. Asterisks indicate non-specific bands. (B) Increasing doses of myc-tagged 14-3-3γ, 14-3-3η and 14-3-3θ and a constant amount of FLAG-MDA5 were co-transfected into RG-I K/D Huh7 cells. The IFNβ promoter activities driven by the ectopic expression of MDA5 alone with 14-3-3γ, 14-3-3η and 14-3-3θ were detected by dual luciferase assay. Ectopic expressed 14-3-3η significantly increased the IFNβ promoter activities driven by MDA5 expression. *: p<0.05, when compared with MDA5 only. (C) Protein expression levels of figure (B) by immunoblotting. (D) MDA5 alone or MDA5 with increasing doses of myc-tagged 14-3-3γ, 14-3-3η and 14-3-3θ were transfected into the RIG-I K/D Huh7 cells. The transfected cells were subsequently stimulated with 1 μg/mL of poly(I:C) for 3 to 6 hours. The IFNβ promoter activities were detected by dual luciferase assay. *: p<0.05, when compared with MDA5 only at the same time point. #: p<0.05, when IFNβ promoter activities in high and low 14-3-3 expressing cells of the same isoform are compared.

It has been reported that RIG-I bind accessory proteins such as TRIM25 and 14-3-3ε as a virus-inducible complex via its CARDs which facilitate the redistribution to the mitochondria associated membrane (MAM) for MAVS interaction. Due to the similarity in structure between RIG-I and MDA5 and the mutual importance of the N-terminal CARDs, we hypothesized that during RNA virus infections or agonists stimulation, MDA5 may also interact with certain 14-3-3 isoforms through the N-terminal CARDs to activate IFNβ signaling pathway to counteract viral infections. To assess whether any of the 14-3-3 isoforms could interact with MDA5, we co-transfected myc-tagged 14-3-3 isoforms and FLAG-tagged WT MDA5 and stimulated the transfected cells with poly(I:C) in preparation for the co-immunoprecipitation assays (Fig 2A and S2A Fig). Six hours after poly(I:C) transfection, the cell lysates were applied to immunoprecipitation by anti-FLAG antibodies. The amount of myc-tagged 14-3-3 proteins co-recovered with FLAG-tagged MDA5 was assayed by immunoblotting (Fig 2A). We found that 14-3-3γ, 14-3-3η and 14-3-3θ could be co-immunoprecipitated with full-length MDA5 in unstimulated cells (Fig 2A). However, we found that MDA5 was in complex with increasing amounts of 14-3-3η post poly(I:C) stimulation, as revealed by the immunoblot analysis of anti-myc 14-3-3 immunoprecipitation products (Fig 2A).

In order to evaluate whether MDA5-interacting 14-3-3 isoforms serve as positive or negative regulators in the MDA5-dependent signaling, we ectopically co-expressed MDA5 and two different doses of 14-3-3 isoforms (14-3-3γ, 14-3-3η and 14-3-3θ) in Huh7 cells, and the IFNβ promoter activities were determined (Fig 2B–2D). In RIG-I knock-down(K/D) Huh7 cells, of which the IFNβ promoter activities were primarily driven by MDA5-dependent signaling (S2B and S2C Fig), we found that the co-expression of 14-3-3η with MDA5 without other stimuli could readily enhance the activation of IFNβ promoter activity in a dose-dependent manner (Fig 2B). The protein expression levels of each constructs were examined by immunoblotting (Fig 2C). With the ability to interact with MDA5, 14-3-3γ and 14-3-3θ, however, did not show an activity to enhance the MDA5-depedent IFNβ promoter activities in either doses tested (Fig 2B and 2C). We further investigated the role of 14-3-3γ, 14-3-3η and 14-3-3θ in promoting the MDA5-dependent IFNβ promoter activities after poly(I:C) stimulation. We found that with the ectopic expression of 14-3-3η in RIG-I K/D Huh7 cells, the IFNβ promoter activities were significantly higher than the control in response to poly(I:C) stimulation (Fig 2D). Similarly, the enhancement by ectopic 14-3-3η to the poly(I:C)-induced MDA5-dependent IFNβ signaling was in a dose-dependent manner of 14-3-3η expression (Fig 2D). In contrast, although ectopic expressions of high amount of 14-3-3γ and 14-3-3θ could slightly induce IFNβ promoter activities than those in the control, these 2 isoforms could not further induce IFNβ promoter activity when high levels of these isoforms were ectopically expressed (Fig 2D). These data suggested that three different 14-3-3 isoforms which could interact with MDA5 might play different roles in MDA5-dependent signaling pathway. Moreover, these results indicated that 14-3-3η could facilitate the activity of MDA5-dependent signaling in upon foreign dsRNA stimulation.

### The depletion of 14-3-3η impairs MDA5 membrane-redistribution, oligomerization, and activation

**Fig 3:**
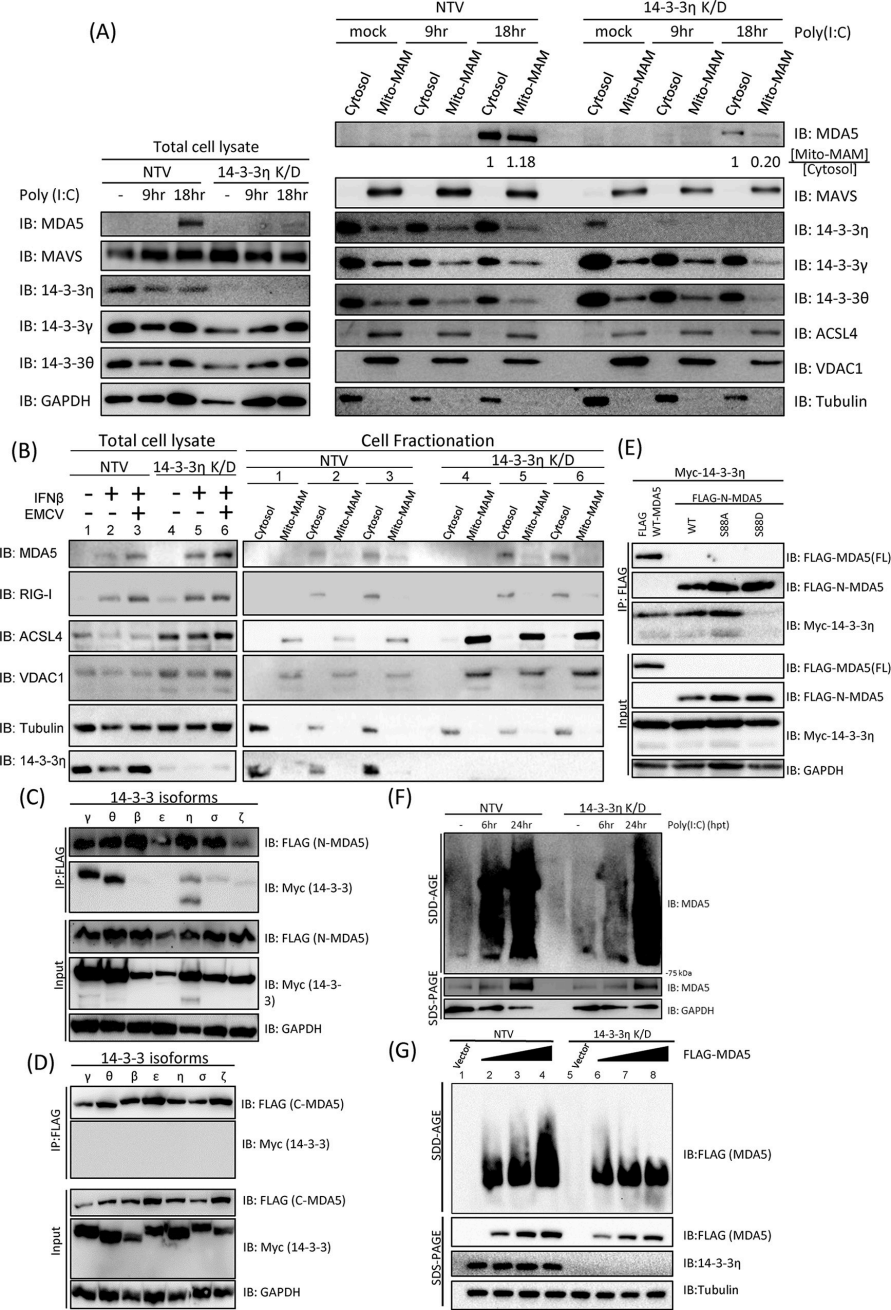
14-3-3η controls MDA5 activation through facilitating MDA5 oligomerization. (A) NTV and 14-3-3η K/D cells were mock treated or stimulated with 1 μg/mL poly(I:C) at different time points. Cell lysates were then fractionated into cytosol or mito-MAM fractions, and the distribution of endogenous MDA5 was monitored by immunoblotting. (B) The NTV and 14-3-3η K/D Huh 7 cells were mock treated or stimulated with 100IU/ml IFNβ for 8 hours followed by EMCV infection at M.O.I of 0.3 for 16 hours. Cell lysates were then fractionated into cytosol or mito-MAM fractions, and the distribution of endogenous MDA5 and RIG-I were monitored by immunoblotting. (C) FLAG-tagged N-MDA5 and different isoforms of myc-tagged 14-3-3 proteins were respectively co-transfected into Huh7 cells. The cell lysates were subject to anti-FLAG immunoprecipitation. 14-3-3γ, 14-3-3η and 14-3-3θ were detected in co-recovered products of immunoprecipitation by immunoblotting. (D) FLAG-tagged C-MDA5 and different isoforms of myc-tagged 14-3-3 proteins were respectively co-transfected into Huh7 cells. The cell lysates were subject to anti-FLAG immunoprecipitation. The recovered proteins by immunoprecipitation were analyzed by immunoblotting. None of the 14-3-3 isoforms were found to interact with FLAG-C-MDA5. (E) Full-length FLAG-tagged MDA5 (MDA5-FL) or FLAG-tagged N-MDA5 with point mutations (S88A and S88D) were co-transfected into HEK293 cells with myc-tagged 14-3-3η. Cell lysates were immunoprecipitated with anti-FLAG beads and the interactions between different MDA5 mutants and 14-3-3η were detected by immunoblotting. (F) NTV and 14-3-3η K/D Huh7 cells were first treated with IFNβ at a concentration of 100 IU/mL for 3 hours and subsequently stimulated with poly(I:C) for 6 or 24 hours. The cells were lysed with triton X-100 lysis buffer and analyzed with SDD-AGE for the aggregation of MDA5 in response to poly(I:C) transfection. The total protein levels of MDA5 from different time points were analyzed by anti-MDA5 immunoblotting after SDS-PAGE. (G) Increasing amount of FLAG-MDA5 plasmids were transfected into NTV and 14-3-3η K/D Huh7 cells respectively. The cells were lysed with triton X-100 lysis buffer and analyzed with SDD-AGE for the aggregation of FLAG-MDA5. The total protein levels of FLAG-MDA5 from different samples were analyzed by anti-FLAG immunoblotting after SDS-PAGE.

Our data suggested that 14-3-3η was a positive regulator of MDA5 activation and the downstream type I IFN induction pathway. We next determined the molecular mechanisms how 14-3-3η could control MDA5 activation. A stable clone of Huh7 cells expressing 14-3-3η targeting shRNA, 14-3-3η K/D Huh7 C#4, was generated for further experiments (S2A Fig). We first assessed the distribution of endogenous MDA5 in NTV or 14-3-3η K/D cells post poly(I:C) stimulation. We found that the redistribution of MDA5 from cytosol to the Mito-MAM fraction in 14-3-3η K/D cells was strongly attenuated when compared to the control NTV Huh7 cells (Fig 3A). The ratio between cytosol MDA5 and Mito-MAM MDA5 is significantly reduced in the 14-3-3η K/D cells when compared to that in the NTV Huh7 cells (Fig 3A). MDA5 has been shown to be an interferon stimulated gene (ISG), of which the expression level increases in response to the presence of interferon. Indeed, the protein level of endogenous MDA5 in NTV Huh7 cells was increased after poly(I:C) stimulation (Fig 3A). However, in the 14-3-3η K/D cells, the endogenous protein level of MDA5 was barely increased post poly(I:C) stimulation when compare to the control NTV Huh7 cells (Fig 3A). To minimize the effects of endogenous MDA5 expression levels in the NTV and 14-3-3η K/D Huh7 cells, the cells were first treated with IFNβ to increase MDA5 expression levels and then infected with encephalomyocarditis virus (EMCV), of which the viral RNA has been reported to specifically serve as ligands of MDA5 to induce MDA5-dependent signaling pathway. IFNβ-containing DMEM was removed right before EMCV infection. In both NTV and 14-3-3η K/D Huh7 cells, we could detect an increase in MDA5 protein expression levels in the total cell lysates, suggesting that the expression levels of 14-3-3η did not affect the induction of MDA5 expression in response to the stimulation of IFNβ (Fig 3B). Previous reports have shown that EMCV replication was highly sensitive to type I IFN treatment [29], and therefore, we first determined whether EMCV entry was diminished by pretreatments of IFNβ by measuring the intracellular EMCV RNA levels (S2 and S3C Figs). We found that although the entry was greatly reduced at the IFNβ pretreatment dose we used (100 IU/ml), EMCV was still able to replicate viral RNA in these cells (S2B and S3C Figs). Also, comparable intracellular EMCV RNA levels, which are the ligands specific for MDA5, were detected in mock- or IFNβ-pretreated NTV and 14-3-3η K/D Huh7 cells after adsorption (S3C Fig). At 18-hour post-infection, the mock- or IFNβ-pretreated NTV and 14-3-3η K/D Huh7 cells lysed and subjected to Mito-MAM fractionation. We found that IFNβ treatment could increase endogenous MDA5 as well as RIG-I protein expressions in the total cell lysates, and the intracellular distribution of RIG-I was not affected by either IFNβ treatment nor EMCV infection (Fig 3B). MDA5 Mito-MAM redistribution was observed in the EMCV-infected NTV cells, and in the 14-3-3η K/D cells, although the MDA5 expression levels were comparable, the Mito-MAM redistribution of MDA5 was marginal (Fig 3B). As a control, the NTV and 14-3-3η K/D Huh7 cells were infected with SeV to assess whether RIG-I activation and Mito-MAM redistribution during viral infection would be affected by 14-3-3η (S3 Fig). We found that the redistribution of RIG-I during SeV infections in the NTV and 14-3-3η K/D Huh7 cells were similar (S3D Fig).

We next assessed prior to the activation-dependent redistribution of MDA5, how 14-3-3η may control MDA5 redistribution. First, we investigated which domain of MDA5 was required for interactions with these 14-3-3 isoforms by co-expressing myc-tagged 14-3-3 proteins with whether FLAG-N-MDA5 or FLAG-C-MDA5 (Fig 3C and 3D). The co-immunoprecipitation results suggested that the N-terminus CARDs of MDA5 had interactions with 14-3-3γ, 14-3-3η and 14-3-3θ and interactions with 14-3-3σ and 14-3-3ζ to some extent. On the other hand, none of the 14-3-3 isoforms had association with C-terminus helicase region of MDA5 (Fig 3C and 3D).

Previous reports have shown that after ligand binding, MDA5 undergoes a dephosphorylation event at S88 for its activation [4]. We first confirmed the activities in inducing IFNβ promoter activities by ectopic expression of MDA5, N-MDA5, and N-MDA5 with S88A or S88D substitutions. As expected and consistent with previous reports, MDA5, N-MDA5 WT, and N-MDA5 S88A could all induce IFNβ promoter activities, and N-MDA5 S88D could not (S3E Fig) [4]. We then assessed whether the interaction between 14-3-3η and MDA5 occurred before or after the dephosphorylation of S88 during MDA5 activation (Fig 3E). Here we again observed that both WT MDA5 and N-MDA5 WT could interact with 14-3-3η (Fig 3E). The S88A mutant of N-MDA5 was able to pull-down comparable amount of 14-3-3η, which is in consistence with previous studies, showing that S88A MDA5 mutant maintained a similar capacity to induce the IFN-β promoter as compared with wild type MDA5. However, only very little or none 14-3-3η could be pulled-down by S88D MDA5 mutants, suggesting that S88 in the N-terminus of MDA5 has the crucial role in the activation of MDA5 as well as 14-3-3 protein interactions (Fig 3E), and the interaction between MDA5 and 14-3-3η may be correlated with MDA5-induced antiviral activities.

According to previous study [12], it has been shown that MDA5 could form oligomers to trigger the activation and interaction between down-stream adaptor protein MAVS and MDA5 itself. We therefore utilized the semi-denaturing detergent agarose gel electrophoresis (SDD-AGE) to detect the aggregation formation of MDA5 in NTV and 14-3-3η K/D cells post poly(I:C) stimulation (Fig 3F). NTV or 14-3-3η K/D cells were first treated with IFNβ to induce the basal protein expression of endogenous MDA5 for 3 hours, and then these cells were stimulated with poly(I:C) for 6 or 24 hours. The kinetics of MDA5 aggregation was delayed in 14-3-3η K/D cells compared to that in the NTV cells post poly(I:C) stimulation (Fig 3F). We next determine the aggregation formation of ectopically expressed FLAG-MDA5 in the NTV and/or 14-3-3η K/D Huh7 cells by SDD-AGE (Fig 3G). The ectopically expressed FLAG-MDA5 were at comparable levels in the NTV andr 14-3-3η K/D Huh7 cells. In the NTV cells, the oligomerization levels of MDA5 was enhanced with the increasing protein levels of FLAG-MDA5; however, although the ectopic expressions of FLAG-MDA5 were at the similar levels, the aggregation formation of MDA5 was much impaired in the 14-3-3η K/D cells (Fig 3G). All these data suggest that 14-3-3η may change/decrease the threshold of innate immunostimulatory potential of foreign viral dsRNA thus to promote MDA5 oligomerization and the redistribution of MDA5 from the cytosol to the Mito-MAM fraction.

### 14-3-3η facilitates the activation of MDA5-dependent antiviral innate immunity

**Fig 4:**
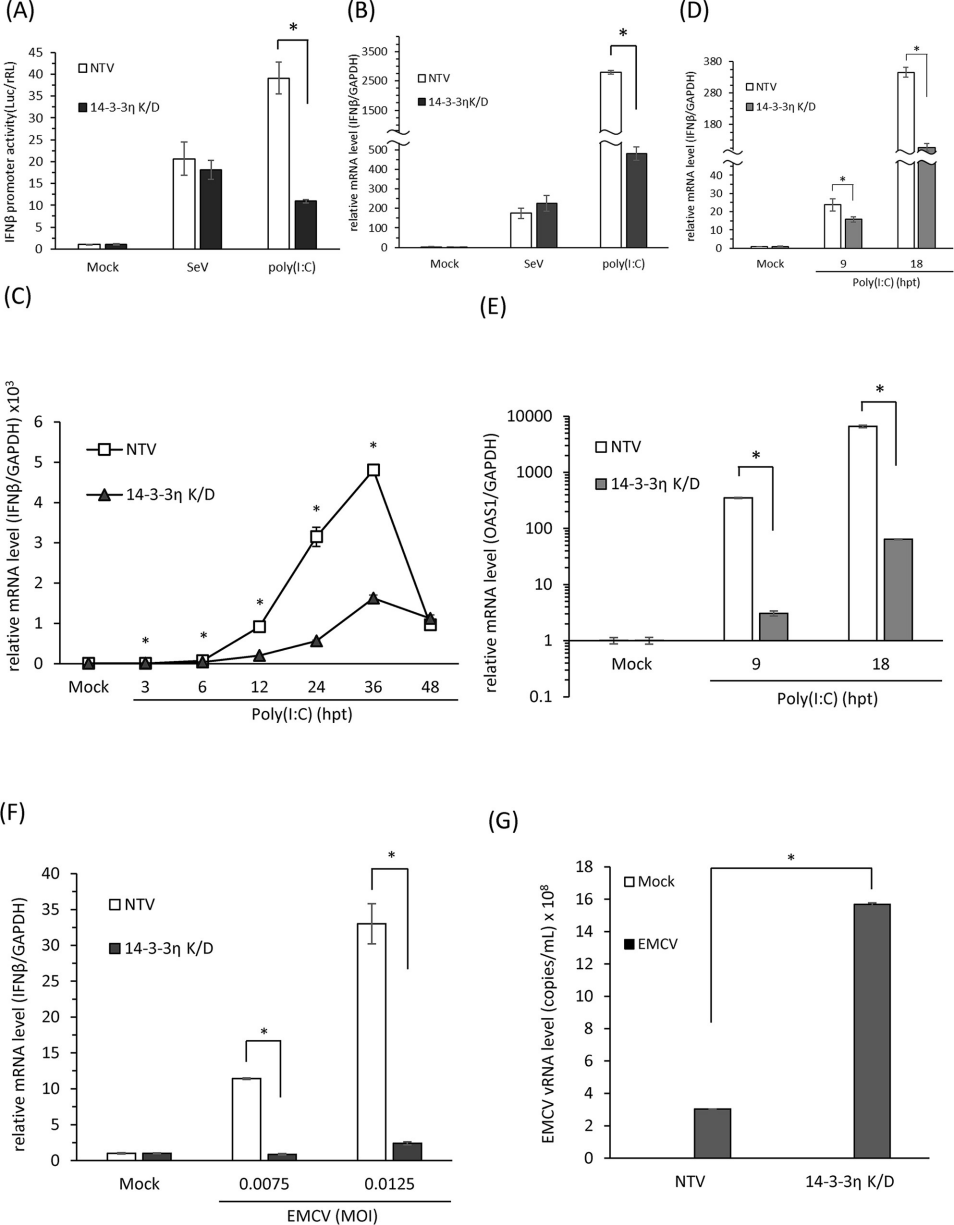
14-3-3η is a critical chaperone protein for MDA5-mediated antiviral activity. (A) IFNβ promoter activities of NTV and 14-3-3η K/D Huh7 cells during SeV infection or poly(I:C) stimulation. NTV and 14-3-3η K/D Huh7 cells were first transfected with pIFNβ-Luc, pCMV-rRL reporter plasmids and subsequently infected by SeV or simulated with poly (I:C) for 18 hours. The promoter activities of IFNβ were evaluated by dual luciferase assay. (B) NTV and 14-3-3η K/D Huh7 cells were mock-treated, infected with SeV, or transfected with poly(I:C) to monitor the expressions of IFNβ mRNA in these cells by quantitative RT-PCR. (C) NTV and 14-3-3η K/D cells were mock-transfected or transfected with 1 μg/mL poly(I:C) for 3 to 48 hours. The intracellular RNA from these cells was extracted and subsequently the IFNβ mRNA expression levels post poly(I:C) stimulation were determined by quantitative RT-PCR. (D and E) NTV or 14-3-3η K/D Huh7 cells were mock-treated or stimulated with poly(I:C) for 9 or 18 hours. The IFNβ and OAS1 mRNA expression levels were determined by quantitative RT-PCR. (F) NTV and 14-3-3η K/D Huh7 cells were mock-treated or infected with EMCV with different M.O.I. for 18 hours. The intracellular RNA from these cells was extracted and the IFNβ mRNA expression levels post EMCV infections were evaluated by quantitative RT-PCR. (*: p<0.05) (G) NTV and 14-3-3η K/D Huh7 cells were mock-treated or infected with EMCV for 18 hours. The intracellular EMCV viral RNA levels was determined by quantitative RT-PCR. (*: p<0.05).

To verify if the 14-3-3η/MDA5 complex is critical for MDA5-dependent type I IFN induction and antiviral activities, we evaluated whether the depletion of 14-3-3η could affect MDA5-dependent signaling pathway. We first evaluated the induction of IFNβ promoter activity during Sendai virus (SeV) infection or poly(I:C) stimulation in NTV and/or 14-3-3η K/D cells. IFNβ promoter activities were induced at comparable levels by SeV infection between NTV and 14-3-3η K/D cells; however, the induction levels of IFNβ promoter activities were significantly decreased in the 14-3-3η K/D cells post poly(I:C) stimulation, compared to those in the NTV cells (Fig 4A). These data together with data from the fractionation assays indicated that loss of 14-3-3η would specifically affect the activation of MDA5-dependent but not the RIG-I-dependent antiviral activity (Figs 3B and 4A and S3 Fig). We also detected the induction of IFNβ mRNA expressions in the NTV and 14-3-3η K/D cells by SeV infection or poly(I:C) stimulation (Fig 4B). A similar phenotype was observed that post poly(I:C) stimulation, the induction of IFNβ mRNA expression was strongly attenuated in the 14-3-3η K/D cells when compared to the NTV cells (Fig 4B). We next stimulated NTV and 14-3-3η K/D cells with poly(I:C) and performed a time-course study of the mRNA expression levels of IFNβ and other ISGs to investigate whether the depletion of 14-3-3η would lead to delayed activation of MDA5-dependent signaling pathway (Fig 4C–4E).

The significant inductions of the IFNβ mRNA expression in both NTV and 14-3-3η K/D cells were first observed at 3 hours post poly(I:C) stimulation, and the IFNβ mRNA levels in both cell lines peaked by 36 hours post poly(I:C) stimulation (Fig 4C). With increasing time post poly(I:C) stimulation, the differences between the levels of induced IFNβ mRNA in NTV and 14-3-3η K/D cells were gradually increased up to 36 hours of poly(I:C) stimulation (Fig 4C). The difference between mRNA expression levels became insignificant at 48 hours of poly(I:C) stimulation (Fig 4C). An independent experiment was performed to observe the mRNA expression levels of IFNβ and other ISGs after poly(I:C) stimulation. Similarly, we observed an impaired IFNβ mRNA induction in the 14-3-3η K/D cells when compared to the NTV cells at the same time point (Fig 4D). Besides, the induction of OAS1 mRNA, an interferon stimulated gene, was also strongly attenuated in 14-3-3η K/D cells in response to poly(I:C) stimulation (Fig 4E). Since the poly(I:C) we used in these experiment primarily induced MDA5-dependent signaling rather than RIG-I-dependent signaling (S2B Fig), these data indicate that 14-3-3η has a critical role in controlling the kinetics of MDA5-dependent type I IFN induction.

Lastly, to determine the critical role of 14-3-3η in the MDA5-dependent type I IFN induction and antiviral activities, we infected the cells with EMCV to determine the activation of MDA5 in 14-3-3η K/D cells (Fig 4F and 4G). After EMCV infection, the production of IFNβ mRNA was significantly decreased in 14-3-3η K/D cells compared to NTV Huh7 cells (Fig 4F). In the NTV cells, we found that the higher M.O.I. of EMCV was introduced, the higher the induction of IFNβ mRNA was detected (Fig 4F). Nevertheless, in 14-3-3η K/D cells, the induction of IFNβ mRNA was drastically reduced in both M.O.I. tested (Fig 4F). We then also analyzed the EMCV vRNA levels in the infected NTV and/or 14-3-3η K/D cells. We found that EMCV vRNA levels were significantly higher in the 14-3-3η K/D cells than those in the NTV cells (Fig 4G). These data together suggest that 14-3-3η indeed has an essential role in the MDA5-dependent antiviral activity. These results indicate that 14-3-3η interaction with MDA5 serves to activate robust MDA5-dependent antiviral activities and innate immune signaling actions.

## Discussion

Previous studies have reported that the activation of RIG-I signaling acquires certain accessory proteins, such as the E3 ubiquitin ligase TRIM25 and the mitochondrial chaperone protein 14-3-3ε [7, 30]. In addition, the C-terminus of RIG-I which contains repressor domain (RD) has the ability to inhibit RIG-I auto-activation [6]. As to MDA5, PP1α/β and RNF125 have been reported to post-translationally modify the CARDs of MDA5 for MDA5 activation [4, 31]. Also, it has been shown that the CARDs of MDA5 oligomerize in response to the interaction of MDA5 and its RNA ligands [12]. The excessive MDA5 accumulation in the cytoplasm may lead to the spontaneous self-association and trigger the oligomerization of CARDs.

According to previous reports, it has been shown that a member within double-stranded RNA-specific adenosine deaminase, ADAR1, serves as an RNA-editing protein that can change the structures of 3’UTR mRNAs to prevent MDA5-autorecognizing reaction. Through changing the secondary structures of UTRs within many mRNAs, ADAR1 enhances the innate immunostimulatory potential of endogenous transcripts thus to reduce the possibilities that MDA5 recognizes self-RNA to activate type I interferon signaling pathway and causes auto-inflammatory diseases[32]. Here we suggest that 14-3-3η may play an opposite role from ADAR1 in host cells to reduce the threshold of MDA5-dependent antiviral signaling pathway activation. 14-3-3η can boost and accelerate the activation of MDA5 signaling that helps host cells establishing well antiviral response as soon as possible to prevent virus infections.

Our data indicated that 14-3-3 proteins could only interact with MDA5 through N-terminus but not the C-terminal domain. Therefore, it is likely that 14-3-3η affects the activation of MDA5 through the interaction between MDA5 N-terminus with no influence the dsRNA binding affinity of MDA5. We analyzed the amino acid sequences of both the CARDs of RIG-I and MDA5 in search of the consensus binding motif of 14-3-3 family. Two modes of consensus phosphor-serine/phosphor-threonine dependent 14-3-3 protein binding motifs were described: R[SFYW]XpSXP (mode 1) and RX[SYFWTQAD]Xp(S/T)X[PLM] (mode 2) (Muslin, A.J., et al. 1996). Neither modes were found within RIG-I CARDs; in contrast, within the amino acid 84 to 90 of MDA5, the R-R-T-G-pS-P-L sequence may serve as the binding motif of 14-3-3 proteins. We initially thought that the S88 phosphorylation of MDA5 may play a role in the binding preference of 14-3-3 isoforms. However, our data showed that the MDA5 S88A mutant served as a better binding partner to 14-3-3η than the S88D mutant, indicating that MDA5 may bind to 14-3-3η in an S88 dephosphorylation-dependent manner. On the other hand, during poly(I:C) stimulation or EMCV infection, we did not observe obvious redistribution of 14-3-3η. This phenomenon was also observed in the RIG-I chaperone, 14-3-3ε [7]. Due to the fact that the 14-3-3 family is a highly conserved chaperone protein family to regulate the intracellular localization of their target proteins, these proteins are likely shuttling around and would be hard to determine the distribution ratio of the 14-3-3 proteins.

Each of the monomer in 14-3-3 contains a binding groove to recognize phosphoserine or phosphothreonine motif. The interaction with 14-3-3 not only may regulate the intracellular localization of proteins but also may stabilize multi-protein complex [33]. In recent reports, phosphoserine- and/or phosphothreonine-independent interaction with 14-3-3 proteins has also been described [34]. For example, the interaction between 14-3-3 proteins and RIG-I CARD domain, exozyme S cytotoxin or phage Raf inhibitor R18 peptide have been found to be phosphorylation-independent [7, 35, 36]. Whether MDA5/14-3-3η interaction is indeed phosphothreonine/phosphoserine independent remains to be further investigated.

**Fig 5:**
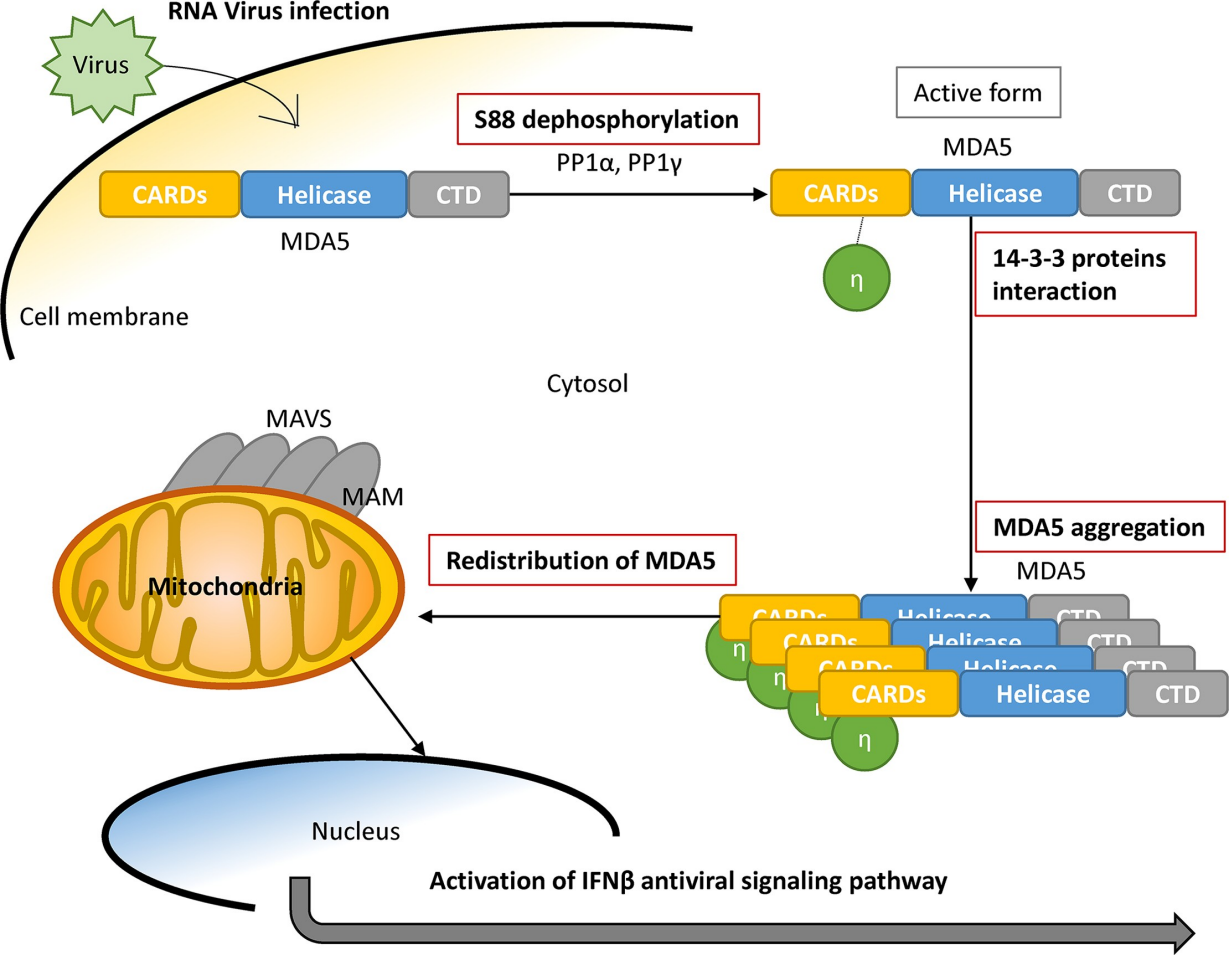
Illustration the proposed model of MDA5 activation.

Several viral proteins have been suggested to interact with 14-3-3, such as HCV core and Parainfluenza Virus 5 M proteins [28, 37]. A recently proteomics report showed that influenza NS1 protein could interact with several 14-3-3 isoforms including 14-3-3γ, 14-3-3η, 14-3-3θ, 14-3-3β and 14-3-3ε, though the effects of NS1 on 14-3-3 proteins remain unclear [38]. These viral protein-14-3-3 interactions may change the typical distribution of 14-3-3 proteins and disrupt the original functions of 14-3-3 family, including to serve as intracellular chaperone proteins regulating the localization of their target proteins. For example, DENV NS3 protein utilizes a phosphomimetic-based mechanism to compete 14-3-3ε in cytosol and thus blocks RIG-I redistributing from cytosol to the mitochondria-associated membrane during DENV infection to impair RIG-I-dependent type I IFN induction [16]. It is suggested that a recombinant mutant DENV deficient in 14-3-3ε binding activates RIG-I-dependent type I IFN induction and might serve as an attenuated vaccine candidate [16]. Here we report that 14-3-3η facilitates MDA5 activation, and it is intriguing to understand whether RNA viruses have developed means to interfere the interaction between 14-3-3η and MDA5 to counteract antiviral activities in the infected cells. Nevertheless, the importance of how each 14-3-3 isoforms may regulate antiviral signaling pathway has arisen in the field of antiviral innate immunity. In summary, our work has defined 14-3-3η as a key component to promote MDA5 activation required for innate antiviral immunity (Fig 5).

## Supporting information

S1 Fig (A) The schematic figure of different MDA5 constructs which used in Figs 1 and 3. (B) Different 14-3-3 protein expression levels and distribution in Fig 1D.
(TIFF)

S2 Fig (A) Overexpression of myc-tagged 14-3-3 isoforms. Different myc-tagged 14-3-3 isoforms were expressed in WT Huh7 cells, and the distinct molecular weight of these 14-3-3 proteins was shown by immunoblotting. (B) IFNβ promoter activities of NTV and RIG-I K/D Huh7 cells post SeV infection or poly(I:C) stimulation. NTV or RIG-I K/D Huh7 cells were first infected with SeV for 18 hours or stimulated with poly(I:C) for 6 hours and the IFNβ promoter activities were detected by dual luciferase assay. (C) The endogenous RIG-I and MDA5 expression levels of NTV and RIG-I K/D Huh7 cells in (B).
(TIFF)

S3 Fig (A) The schematic figure which describes the selection of 14-3-3η knock-down Huh7 stable cells. Huh7 cells were transfected with 14-3-3η-targeting shRNA and the puromycin-resistant colonies were selected. The endogenous 14-3-3η expression level of each colony was determined by immunoblotting. For later experiments in these study, 14-3-3η K/D Huh7 cells #4 were used. (B, C) NTV and 14-3-3η K/D Huh7 cells were treated with IFNβ (100 IU/mL) for 8 hours, and were subsequently infected with EMCV for 1 or 18 hours. Total RNA of these cells were extracted and viral RNA copies of EMCV were evaluated with real-time PCR. The presence of EMCV vRNA could be detected post IFNβ stimulation in both NTV and 14-3-3η K/D Huh7 cells. (D) The NTV and 14-3-3η K/D Huh 7 cells were mock treated or infected with SeV for 16 hours. Cell lysates were then fractionated into cytosol or mito-MAM fractions, and the distribution of endogenous MDA5 and RIG-I were monitored by immunoblotting. (E) The IFNβ promoter activities which induced by different MDA5 constructs and mutants. HEK293 cells were first transfected with different FLAG-tagged MDA5 constructs and pIFNβ-Luc, pCMV-rRL for 48 hours. The promoter activities of IFNβ were evaluated by dual luciferase assay. Protein expression levels were detected by immunoblotting.
(TIFF)
