# Supplementary figures and images for: The 14-3-3η chaperone protein promotes antiviral innate immunity via facilitating MDA5 oligomerization and intracellular redistribution
Source: PLoS Pathog. 2019 Feb 11;15(2):e1007582. doi: 10.1371/journal.ppat.1007582 (PMC6386420; doi:10.1371/journal.ppat.1007582)

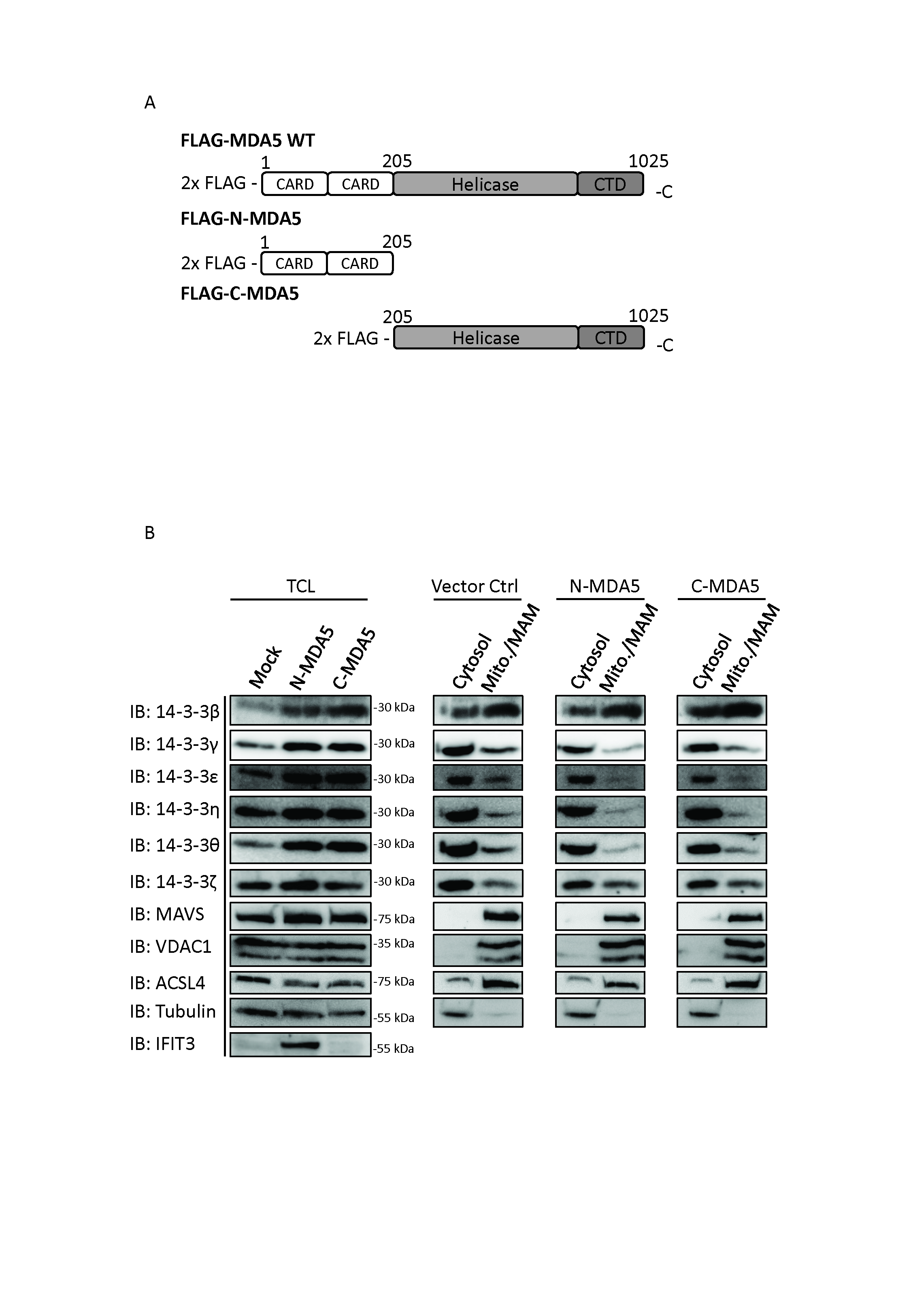

Supplement: S1 Fig — (A) The schematic figure of different MDA5 constructs which used in Figs 1 and 3. (B) Different 14-3-3 protein expression levels and distribution in Fig 1D. (TIFF) [file ppat.1007582.s001.tiff]

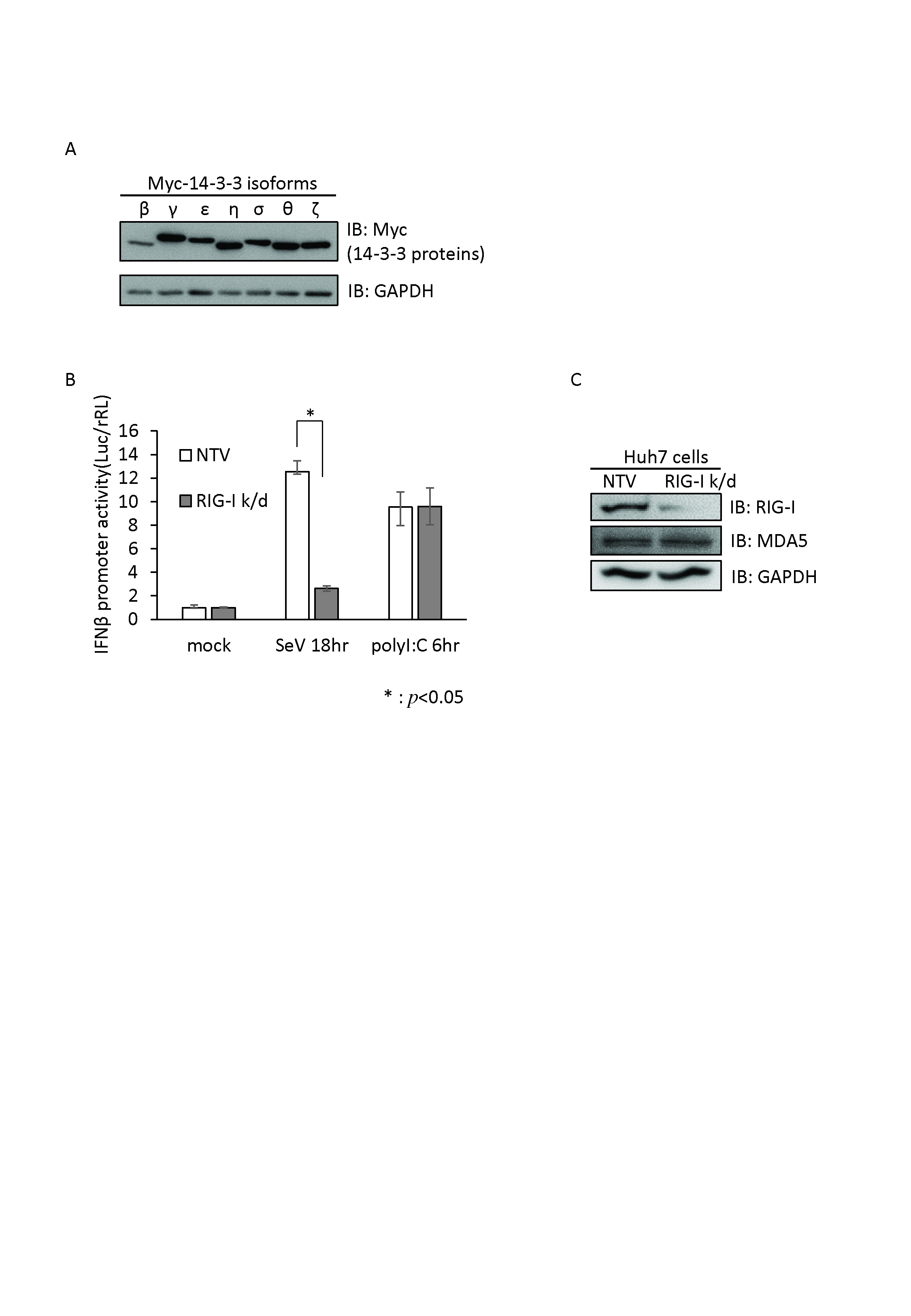

Supplement: S2 Fig — (A) Overexpression of myc-tagged 14-3-3 isoforms. Different myc-tagged 14-3-3 isoforms were expressed in WT Huh7 cells, and the distinct molecular weight of these 14-3-3 proteins was shown by immunoblotting. (B) IFNβ promoter activities of NTV and RIG-I K/D Huh7 cells post SeV infection or poly(I:C) stimulation. NTV or RIG-I K/D Huh7 cells were first infected with SeV for 18 hours or stimulated with poly(I:C) for 6 hours and the IFNβ promoter activities were detected by dual luciferase assay. (C) The endogenous RIG-I and MDA5 expression levels of NTV and RIG-I K/D Huh7 cells in (B). (TIFF) [file ppat.1007582.s002.tiff]

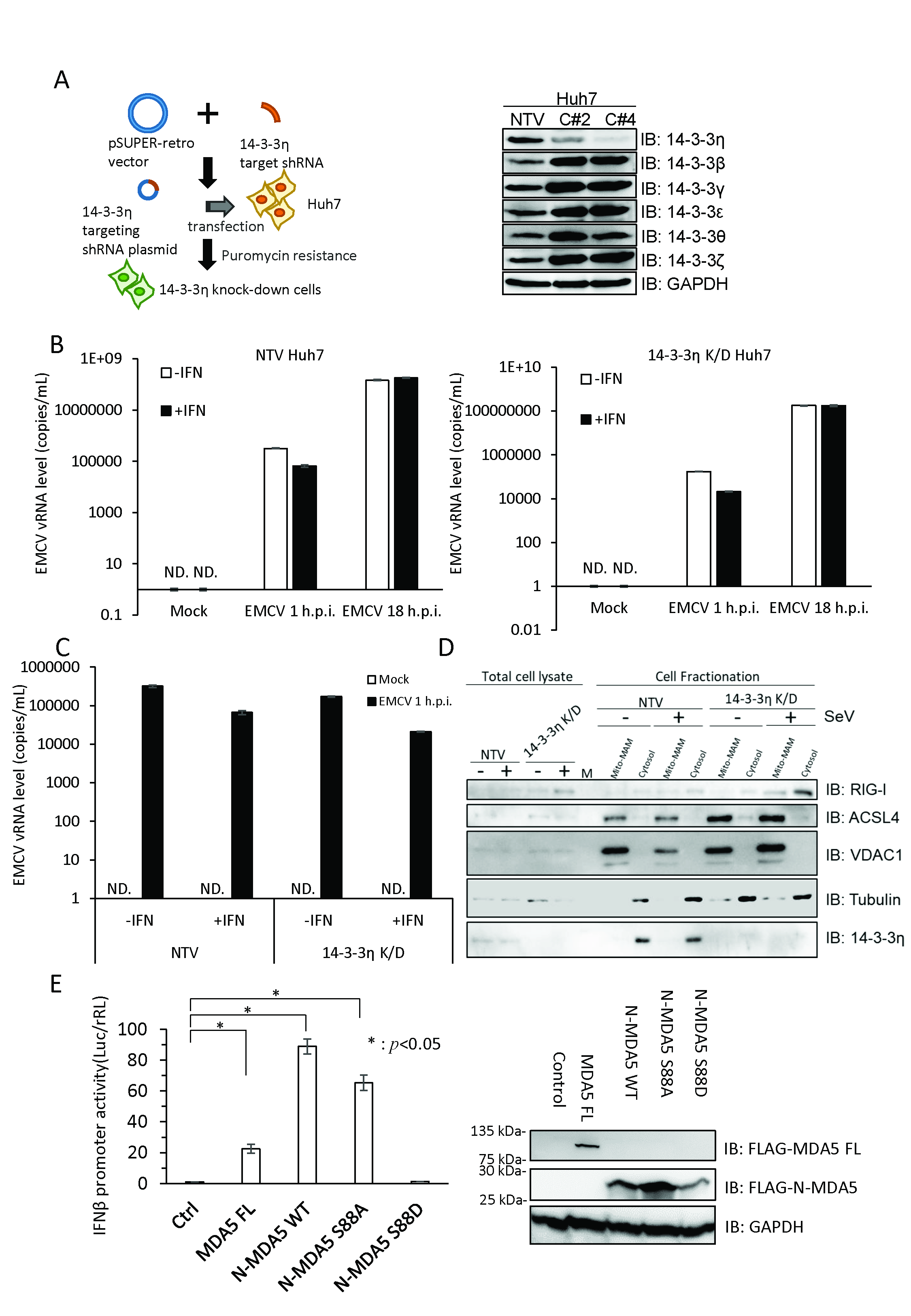

Supplement: S3 Fig — (A) The schematic figure which describes the selection of 14-3-3η knock-down Huh7 stable cells. Huh7 cells were transfected with 14-3-3η-targeting shRNA and the puromycin-resistant colonies were selected. The endogenous 14-3-3η expression level of each colony was determined by immunoblotting. For later experiments in these study, 14-3-3η K/D Huh7 cells #4 were used. (B, C) NTV and 14-3-3η K/D Huh7 cells were treated with IFNβ (100 IU/mL) for 8 hours, and were subsequently infected with EMCV for 1 or 18 hours. Total RNA of these cells were extracted and viral RNA copies of EMCV were evaluated with real-time PCR. The presence of EMCV vRNA could be detected post IFNβ stimulation in both NTV and 14-3-3η K/D Huh7 cells. (D) The NTV and 14-3-3η K/D Huh 7 cells were mock treated or infected with SeV for 16 hours. Cell lysates were then fractionated into cytosol or mito-MAM fractions, and the distribution of endogenous MDA5 and RIG-I were monitored by immunoblotting. (E) The IFNβ promoter activities which induced by different MDA5 constructs and mutants. HEK293 cells were first transfected with different FLAG-tagged MDA5 constructs and pIFNβ-Luc, pCMV-rRL for 48 hours. The promoter activities of IFNβ were evaluated by dual luciferase assay. Protein expression levels were detected by immunoblotting. (TIFF) [file ppat.1007582.s003.tiff]
